# The Assessment of Serum Concentrations of AGEs and Their Soluble Receptor (sRAGE) in Multiple Sclerosis Patients

**DOI:** 10.3390/brainsci11081021

**Published:** 2021-07-31

**Authors:** Aleksandra Damasiewicz-Bodzek, Beata Łabuz-Roszak, Bartłomiej Kumaszka, Bartosz Tadeusiak, Krystyna Tyrpień-Golder

**Affiliations:** 1 Department of Chemistry, School of Medical Sciences in Zabrze, Medical University of Silesia, 40-055 Katowice, Poland; bartosztadeusiak@gmail.com (B.T.); ktyrpien@sum.edu.pl (K.T.-G.); 2 Department of Neurology, Institute of Medical Sciences, University of Opole, 45-040 Opole, Poland; blabuzroszak@gmail.com; 3 J. Glowatzki Hospital, 47-100 Strzelce Opolskie, Poland; b.kumaszka@op.pl; 4 City Hospital No. 4, 44-100 Gliwice, Poland

**Keywords:** advanced glycation end products, AGE, RAGE, sRAGE, multiple sclerosis, ELISA

## Abstract

Background: Advanced glycation end products (AGEs) are involved in the pathogenesis of many diseases, including neurodegenerative diseases such as multiple sclerosis (MS). The aim of the study was to determine serum concentrations of AGEs and their soluble receptor (sRAGE) in MS patients and healthy controls and to investigate their possible influence on disease activity. Methods: Serum concentrations of AGE and sRAGE in patients with MS and healthy controls were determined by enzyme-linked immunosorbent assay (ELISA). Results: The mean serum AGE concentration in patients with MS was higher than in healthy controls, whereas the mean serum sRAGE concentration was lower than in the control group. However, the differences were not statistically significant. In MS patients, serum AGE and sRAGE concentrations did not differ significantly, depending on the duration of the disease and the Expanded Disability Status Scale (EDSS) score. Conclusions: Multiple sclerosis may be accompanied by disturbances of the AGE-sRAGE axis. However, further studies are warranted to confirm it. The duration of the disease and the degree of disability do not seem to affect the progression of the glycation process, particularly in the stable phase of the disease.

## 1. Introduction

Multiple sclerosis (MS) is a chronic inflammatory demyelinating disease involving the central nervous system [1,2,3,4,5,6,7,8,9], whose etiopathogenesis is not fully understood. Infectious, genetic, environmental, and immune factors are also involved [10,11,12,13,14,15,16,17,18,19,20]. Among environmental factors, the correlations between the increase in the incidence and the increase in latitude in the northern hemisphere as well as decreased incidence in the southern hemisphere were noted [21]. It is probably associated with lower serum vitamin D3 concentrations in MS patients [9,22,23,24,25,26,27]. It has been proven that vitamin D3 prevents autoimmune diseases. Vitamin D3 is a powerful antioxidant that fights free radicals and reduces oxidative stress in the nervous system and prevents MS, reduces its symptoms, and relapses [28]. There are many observations suggesting that the state of increased oxidative stress and intensified production of reactive oxygen species (ROS) is the key bridge between inflammation and neurodegeneration in MS etiopathogenesis. The cellular source of free radical formation largely depends on the stage of the MS lesions [29]. Both activated microglia and infiltrated macrophages are able to generate vast amounts of proinflammatory mediators and oxidizing radicals, such as superoxide, hydroxyl radicals, hydrogen peroxide, and nitric oxide [29,30]. Important sources of oxidizing species are the ROS-generating enzymes: myeloperoxidase, xanthine oxidase, and NADPH oxidase [29,31]. Another source of oxidative stress in the central nervous system are enzymes associated with arachidonic-acid signaling; free radicals are also produced by cyclooxygenases and lipoxygenase [32,33]. Therefore, neuroinflammation can trigger oxidative stress by at least two different mechanisms: production of high levels of ROS by activated glia and activated arachidonic-acid pathways [32,34]. Increased oxidative stress and weakened antioxidant barriers may simultaneously contribute to processes of advanced protein glycation.

Protein glycation is a multistage, non-enzymatic process of protein modification by reducing sugars. It occurs under physiological conditions, but it may also cause pathological processes under certain circumstances [35,36]. The first stage is related to the formation of a Schiff’s base from the carbonyl group of sugar and the amino group of the protein (mostly amine residues of lysine and histidine, arginine, valine, and other amino acids, which are less common). After several weeks, this product undergoes rearrangement known as the Amadori reaction. Next, it undergoes Maillard reactions with the formation of advanced glycation end products (AGEs) [37,38,39]. They include N^ε^-carboxymethyllysine (CML), N^ε^-carboxyethyllysine (CEL), pentosidine, pyraline, imidazoles, and other compounds [40,41,42,43]. These products can also be delivered with food (gliotoxins). AGEs bind to their specific receptors known as RAGE (receptors for AGEs) [44], which are found on the surface of many cells in the body. This connection results in a cascade of reactions involving the increase in the production of cytokines, growth factors, and other proinflammatory compounds [45]. RAGEs are one of the five basic types of membrane receptors. The role of other receptors (such as AGE-R1, AGE-R2, AGE-R3, and MSR1) is to eliminate AGE from the circulation. Other defense mechanisms against protein glycation include lysosomes on the cell surface, degradation of proteasomes, and the humoral response. This response occurs through the production of anti-AGE antibodies in response to too high concentrations of AGEs in chemical compounds, which causes their dysfunction and destruction as a result of the immune reaction [46,47,48].

In addition to membrane receptors, the soluble receptor (sRAGE) is also recognized [49]. It consists of two isoforms: endogenous secretory RAGE (esRAGE) and cleaved RAGE (cRAGE) [50]. Due to their structure, which is different from the RAGE receptor, the presence of sRAGE does not cause an inflammatory response and weakens the AGE response to RAGE [51]. Decreased sRAGE concentration is found in cognitive disorders [52] and in Guillain-Barre syndrome [53]. Inflammation, which results from excessive glycation of proteins, is the cause of many diseases [54]. The significance of AGEs has been proven in the etiopathogenesis of Alzheimer’s disease [55,56,57], Parkinson’s disease [58], and amyotrophic lateral sclerosis (ALS) [59].

The aim of the study was to determine the serum concentrations of AGEs and their soluble receptor (sRAGE) in MS patients and healthy controls and to investigate their possible influence on disease activity.

## 2. Materials and Methods

The study group consisted of patients with MS who were residents of the Province of Silesia and were associated with SEZAM, which is the Silesian Association of MS (Gliwice, Poland). The age-matched control group comprised healthy volunteers. Each MS patient completed the questionnaire related to their history (age, sex, place of residence, onset, duration and course of the disease, degree of disability, quality of life, and treatment). The medical records underwent detailed analysis. Each patient underwent neurological assessment, and the functional status was determined by the Expanded Disability Status Scale (EDSS).

The inclusion criteria in the study group were as follows: age ≥ 18 years, MS diagnosis based on the McDonald criteria (2010), the results of magnetic resonance imaging [60], and informed consent. The exclusion criteria were as follows: neurological comorbidities other than MS (i.e., dementia, previous stroke, neuropathy, cervical radiculopathy, etc.), chronic systemic diseases (i.e., diabetes, advanced heart failure, chronic renal disease, thyroid diseases, autoimmune diseases, etc.), and infectious diseases (especially Lyme disease). The control group consisted of healthy adults with no history of familial neurodegenerative diseases. The study was approved by the Local Bioethics Committee of the Medical University of Silesia, Katowice. All participants were informed and gave written informed consent for study participation.

Fasting blood samples were collected from the elbow vein (7 mL). Serum samples obtained by centrifugation were stored at −85 °C until analysis. Serum concentrations of AGEs and sRAGE in MS patients and healthy volunteers were determined by the enzyme-linked immunosorbent technique (ELISA) using commercially available kits. The OxiSelectTM AGEs ELISA Kit (CELL BIOLABS Inc., USA; catalog number STA-317) was used to determine serum concentrations of AGEs. The RayBio^®^ Human RAGE ELISA Kit (Ray Biotech, Inc., Peachtree Corners, GA, USA) was used to determine sRAGE concentrations in the tested samples. Absorbance readings were created using the Power Wave XS reader (BioTek, Winooski, VT, USA) at a wavelength of 450 nm (reference wave 630 nm), and the results were processed using the KC Junior computer program (BioTek, Winooski, VT, USA). The intra-assay variation was below 10%. The sensitivity of the assays was 0.5 μg/mL for AGE and 3 pg/mL for sRAGE.

The results were presented using the basic parameters of descriptive statistics. The normality of the distribution of variables was tested by the Shapiro–Wilk test. Non-parametric Kolmogorov–Smirnov and Mann–Whitney U tests were used for comparisons between the groups. The Kruskal–Wallis rank ANOVA test was used to study variability in the MS group, while the Spearman rank test was applied for correlations; *p* < 0.05 was considered statistically significant. The calculations were performed using STATISTICA for Windows 12.0 (StatSoft, Cracow, Poland).

## 3. Results

### 3.1. Participants

The study group consisted of 52 patients with MS (35 women and 17 men; mean age 37.9 ± 9.4 years), and the control group comprised 40 healthy volunteers (25 women and 15 men; mean age 41.1 ± 10.4 years). Gender distribution and age were comparable in both groups (*p* = 0.689 and *p* = 0.128, respectively).

MS patients were characterized by different disease duration from the onset of first symptoms: 0–5 years: 29%; 6–10 years: 33%; 11–15 years: 24% and 16 and more years: 14%. Relapsing-remitting MS was diagnosed in 73% of patients, whereas secondary progressive MS was diagnosed in 27% of subjects. The assessment of motor function showed that 40% of patients had an EDSS score ranging from 0 to 1.5 points, 33% of patients from 2 to 4.5 points, and 27% of patients had a score of ≥5 points. In the study group, the most common symptoms of the disease included weakness in at least one limb (76%), balance disorders (50%), mood disorders (42%), sensory disturbances (42%), visual impairment (34%), and bladder problems (21%). Twenty patients were treated with disease-modifying drugs (DMDs) such as interferon beta (*n* = 13), glatiramer acetate (*n* = 2), natalizumab (*n* = 2), and fingolimod (*n* = 3). Interferon beta and glatiramer acetate were the drugs of first choice, patients treated with natalizumab and fingolimod were first treated with interferon. The basic demographic and clinical data of MS patients are presented in Table 1.

### 3.2. Serum AGE and sRAGE Concentrations

Despite the lack of statistical significance, the mean serum AGE concentrations were slightly elevated in MS patients (46.78 ± 9.61 µg/mL vs. 44.65 ± 12.40 µg/mL, Mann–Whitney U test: *p* = 0.134), while the mean serum sRAGE concentrations were slightly decreased in MS patients (331.59 ± 129.17 pg/mL vs. 354.80 ± 171.88 pg/mL, Mann–Whitney U test: *p* = 0.716) compared with the control group. No significant differences were found between men and women in both groups (Table 2).

Serum AGE and sRAGE concentrations did not correlate with age in the MS group (Spearman rank test: R = 0.030, *p* = 0.844 for AGE and R = 0.023, *p* = 0.880 for sRAGE) or in the control group (Spearman rank test: R = 0.027, *p* = 0.883 for AGE and R = −0.255, *p* = 0.139 for sRAGE).

**Figure 1:**
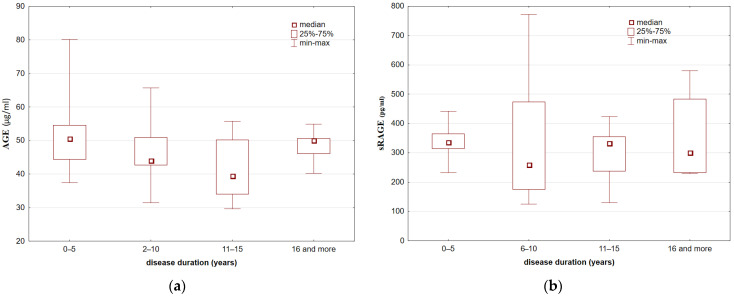
Serum AGE (**a**) and sRAGE; (**b**) concentrations in MS patients depending on disease duration (Kruskal–Wallis rank ANOVA test: *p* > 0.05).

**Figure 2:**
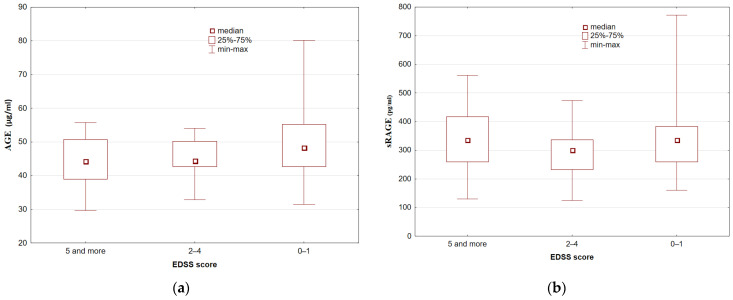
Serum AGE (**a**) and sRAGE (**b**) concentrations in MS patients depending on the EDSS score (Kruskal–Wallis rank ANOVA test: *p* > 0.05).

We found no associations between serum AGE and sRAGE concentrations and disease duration (Kruskal–Wallis rank ANOVA test: *p* = 0.12 and *p* = 0.86, respectively) or between serum AGE and sRAGE concentrations and the EDSS score (Kruskal–Wallis rank ANOVA test: *p* = 0.27 and *p* = 0.38, respectively) (Figure 1 and Figure 2).

In the study group, the mean AGE and sRAGE concentrations did not differ significantly between patients treated and untreated with DMDs (Mann–Whitney U test: *p* = 0.23 and *p* = 0.75, respectively). Moreover, the mean AGEs and sRAGE concentrations were similar in patients treated with interferon β and those treated with other DMDs (Mann–Whitney U test: *p* = 0.86 and *p* = 0.91, respectively).

## 4. Discussion

It is hypothesized that AGEs can cause proinflammatory effects in microglia and astrocytes and can cause blood-brain barrier (BBB) dysfunction [61,62]. Protein glycation and oxidation occur physiologically. However, they cause adverse changes in tissues and organs. It has been shown that AGEs also reach higher concentrations in different neurodegenerative diseases, including Alzheimer’s disease and ALS [63,64,65]. An attempt was also made to assess whether the concentrations of AGEs, anti-AGE antibodies and circulating immune complexes containing AGE could serve as specific biomarkers of neurodegeneration [56,57].

In our study, although the mean serum AGE concentration was higher and the mean serum sRAGE concentration was lower in MS patients, the differences were not statistically significant. Similar conclusions were obtained by Kalousova et al. [66], who did not find statistically significant differences in the concentrations of AGEs or pentosidine between MS patients and the control group in the serum or the cerebrospinal fluid. Those authors reported that the role of AGEs as a glycation marker in MS was limited [67].

In turn, Stenberg et al. [68] noticed that the mean concentration of N^ε^-carboxyethyllysine (CEL) was higher in patients with stable MS compared with the control group. They also reported higher concentrations of another AGE known as N^ε^-carboxymethyllysine (CML) in patients with active MS. Those researchers concluded that serum AGEs, and particularly CEL, might be useful MS biomarkers and suggested the introduction of AGE-inhibitor therapy.

The findings of Gilden et al. were consistent with the above study in terms of the use of AGE inhibitors in MS treatment [10]. It is postulated that angiotensin-converting enzyme inhibitors (ACEIs) can be used [68]. The degradation of AGEs was reported in diabetic patients on ACEIs compared with those who were not on ACEIs [69,70]. Therefore, further research is warranted to confirm the role of AGEs in the etiopathogenesis of MS.

AGEs accumulate in tissues, which can indicate the inflammatory process. Given that the clinical relapse in MS patients usually occurs once or twice annually, and the flare-ups last less than two weeks, the duration of the relapse appears to be insufficient to cause accumulation of AGEs in the blood. In our study, MS patients were in a stable disease phase. Therefore, the time of AGE accumulation may have been too short. Determination of the concentrations of AGEs in the same patient during clinical relapses and remissions may provide a better indication of the usefulness of AGEs as a marker of the risk of subsequent relapses [68].

The secretory form of the RAGE receptor (sRAGE), which is protective against the toxic effects of AGE-RAGE, was also found in the circulation [71]. The role of the sRAGE receptor in the etiopathogenesis of MS was proven by Glanović et al. [72], who characterized the HMGB1/sRAGE axis in MS patients. The authors observed a significantly reduced concentration of sRAGE in the cerebrospinal fluid in MS patients. The authors suggested that sRAGE could become a marker for MS. Stenberg et al. [73] reached similar conclusions as Glanović et al. [72]. The authors noticed a significantly lower serum concentration of sRAGE in MS patients compared to healthy individuals.

Moreover, the results were associated with gender and the severity of the disease (EDSS), but not with disease duration. The authors speculated that serum sRAGE levels were related to the frequency of relapses, as patients with low serum sRAGE levels had a higher annualized relapse rate (ARR) than patients with higher sRAGE levels. The study also found that the mean concentration of sRAGE was lower in women with MS than in men with MS. This tendency may be related to the differences in steroid hormone levels between the genders. In particular, 17β-estradiol, which is a sex hormone, influences the activation of the RAGE receptor [74]. Moreover, women with MS show increased production of proinflammatory cytokines compared to affected men [75], which may partially explain the gender differences in serum sRAGE levels.

The analysis of the literature also includes studies on the influence of the endogenous secretory-RAGE (esRAGE) in the etiopathogenesis of MS. According to Stenberg et al. [75], esRAGE can be used as a biomarker of relapse. Patients with relapsing-remitting MS had lower levels of esRAGE at the time of relapse compared to patients with stable disease. Stenberg et al. suggested that esRAGE concentration was positively modulated by appropriate pharmacotherapy as opposed to sRAGE and indicated that there were differences in esRAGE concentrations depending on the type of MS. Čierny et al. [76] revealed a significantly elevated level of sRAGE in patients with MS compared with the control group. They did not confirm the association between sRAGE concentration and disease disability progression or different types of MS [76].

In our study, we did not observe any differences in serum AGE or sRAGE concentrations between pharmacologically treated and untreated MS patients. Rahimi et al. [77] and Asadikaram et al. [78] noted elevated serum sRAGE levels in patients on interferon β. Moreover, Sternberg et al. [79] observed that fingolimod was also associated with the RAGE axis, which appears to significantly contribute to the anti-inflammatory and neuroprotective effects of fingolimod. One year of treatment with this drug increased serum levels of sRAGE isoforms by 32.4%. Sternberg et al. noted that MS patients receiving immunomodulatory drugs had mean CEL concentration decreased by 40% versus the untreated subjects, but the CEL levels were still significantly higher than in the control group.

In summary, our study was unique in the comprehensive assessment of serum concentrations of AGEs and their soluble receptor (sRAGE) in MS patients depending on age, gender, duration of the disease, degree of disability, and used DMD.

In our opinion, the relationship between advanced protein glycation and MS requires further research due to inconclusive study results. The pathological processes in MS may influence the relationship between selected glycation products. In the future, sRAGE and esRAGE may serve as diagnostic tests for MS, differentiate various types of the disease, and can be used to monitor the risk of relapse. The use of AGE inhibitors, which have a neuroprotective effect, seems to be a solution [80]. Additionally, their soluble receptors in combination with rehabilitation may improve therapeutic effects and improve the quality of life of patients [81].

## 5. Conclusions

MS may be accompanied by disturbances of the AGE-sRAGE axis. However, it should be confirmed by further research. The duration of the disease and the degree of disability do not seem to affect the progression of the glycation process, particularly in the stable phase of the disease.

## 6. Limitations of the Study

The small size of patient groups and subgroups (i.e., treated and untreated with DMDs) may influence statistical differences. Additionally, only clinically stable patients were assessed. It will be interesting to compare serum AGEs and sRAGE levels during relapse and remission in the same patients. Therefore, further studies are warranted to address this issue.

## Figures and Tables

**Table 1:** The basic demographic and clinical data of MS patients.

Characteristics	MS Patients
**N**	52
**Age (years)** mean ± SD	37.9 ± 9.4
**Gender** female (%) male (%)	35 (67%) 17 (33%)
**Employment** full-time employment/student part-time employment pension/sickness pension	28 (54%) 5 (10%) 19 (36%)
**Smoking** active smokers passive smokers non-smokers	8 (15%) 12 (23%) 32 (62%)
**Environmental exposure** chemical pollution/industrial plants heat plants/power plants increased car traffic	25 (48%) 5 (10%) 25 (48%)
**Moving** no problem with the help of elbow crutches on wheelchair	38 (73%) 10 (19%) 4 (8%)
**Forms of physical activity** walking/nordic walking biking none	41 (79%) 4 (8%) 5 (10%)
**Active forms of rehabilitation** every day several times a year once a year every few years never	2 (4%) 25 (48%) 11 (19%) 4 (8%) 10 (19%)
**Disease duration (years)** 0–5 6–10 11–15 16 and more	15 (29%) 17 (33%) 13 (24%) 7 (14%)
**Form of the disease** relapsing-remitting secondary progressive	38 (73%) 14 (27%)
**EDSS score (points)** 0–1.5 2–4.5 5 and more	21 (40%) 17 (33%) 14 (27%)
**The most common symptoms** weakness in at least one limb balance disorders mood disorders sensory disturbances visual impairment bladder problems	40 (76%) 26 (50%) 22 (42%) 22 (42%) 18 (34%) 11 (21%)
**DMD treatment** interferon beta glatiramer acetate natalizumab fingolimod	13 (25%) 2 (4%) 2 (4%) 3 (6%)

**Table 2:** Serum AGEs and sRAGE concentrations in MS patients and healthy controls depending on the gender (Mann–Whitney U test).

Parameter (Mean ± SD)	MS Patients (*n* = 52)			Control Group (*n* = 40)		
	Men (*n* = 17)	Women (*n* = 35)	*p*	Men (*n* = 15)	Women (*n* = 25)	*p*
AGE (µg/mL)	47.75 ± 9.12	46.30 ± 9.96	0.438	43.16 ± 11.39	45.47 ± 13.13	0.555
sRAGE (pg/mL)	303.66 ± 122.03	345.56 ± 132.37	0.322	373.98 ± 167.80	346.00 ± 176.56	0.472

## Data Availability

The data presented in this study are available on request from the corresponding author.
